# Robustness of Radiomics for Survival Prediction of Brain Tumor Patients Depending on Resection Status

**DOI:** 10.3389/fncom.2019.00073

**Published:** 2019-11-08

**Authors:** Leon Weninger, Christoph Haarburger, Dorit Merhof

**Affiliations:** Imaging and Computer Vision, RWTH Aachen University, Aachen, Germany

**Keywords:** BraTS 2018, survival prediction, radiomics, brain tumor, machine learning, feature selection

## Abstract

Prediction of overall survival based on multimodal MRI of brain tumor patients is a difficult problem. Although survival also depends on factors that cannot be assessed via preoperative MRI such as surgical outcome, encouraging results for MRI-based survival analysis have been published for different datasets. We assess if and how established radiomic approaches as well as novel methods can predict overall survival of brain tumor patients on the BraTS challenge dataset. This dataset consists of multimodal preoperative images of 211 glioblastoma patients from several institutions with reported resection status and known survival. In the official challenge setting, only patients with a reported gross total resection (GTR) are taken into account. We therefore evaluated previously published methods as well as different machine learning approaches on the BraTS dataset. For different types of resection status, these approaches are compared to a baseline, a linear regression on patient age only. This naive approach won the 3rd place out of 26 participants in the BraTS survival prediction challenge 2018. Previously published radiomic signatures show significant correlations and predictiveness to patient survival for patients with a reported subtotal resection. However, for patients with reported GTR, none of the evaluated approaches was able to outperform the age-only baseline in a cross-validation setting, explaining the poor performance of approaches based on radiomics in the BraTS challenge 2018.

## 1. Introduction

The high-grade glioma, a subtype of brain tumors, is one of the most aggressive and dangerous diseases worldwide. For the US, a 5-year survival rate of glioblastoma patients of only 5.6% was reported for 2000–2015 (Ostrom et al., 2018). Automatic analysis of these tumors is challenging, as their shape, location and extent can differ substantially. Since 2012, the BraTS challenge (Menze et al., 2015) is held annually to allow an unbiased comparison of different segmentation algorithms. Since 2017, an overall survival (OS) prediction task is included to assess whether quantitative image features based on these segmentations can provide further clinical insight. In the OS task, patients need to be classified in *long-survivors* (OS>15 months), *short-survivors* (OS <10 months), and *mid-survivors* (10 months <OS <15 months). While data is provided for patients with different resection status, the official evaluation is carried out only on patients with a reported gross total resection (GTR). A total of 41 teams took part in this survival prediction task in 2017 and 2018.

Using the age as sole feature with a linear regressor, we achieved an accuracy of 0.56 (*n* = 77) on the test set in the BraTS challenge 2018. In comparison, the first placed approaches of 2017 (Shboul et al., 2018) and 2018 (Feng et al., 2019) achieved accuracies of around 0.58 and 0.62, respectively (Bakas et al., 2018b). Shboul et al. relied on automatic radiomic feature extraction combined with a Random Forest Regressor (RFR), while Feng et al. used geometric features in combination with a linear model. The developers of other top performing algorithms chose similar strategies of combining either hand-selected or automated radiomic features with a supervised machine learning algorithm: Radiomic feature extraction was used in combination with an RFR (Sun et al., 2019) or a Multilayer Perceptron (MLP) (Baid et al., 2019). Geometric features only were used with an MLP (Jungo et al., 2018), and finally atlas locations together with relative tumor sizes and an RFR were also employed (Puybareau et al., 2019). These teams achieved accuracies between 0.55 and 0.6. Further submitted approaches ranged from deep learning algorithms to radiomic feature analysis to handcrafted feature engineering, that achieved accuracies between 0.15 and 0.55. As three classes were equally subdivided, a random choice would result in an accuracy of 0.33.

On other brain tumor datasets, encouraging results for OS prediction have been published. A successful radiomic-based brain tumor patient OS and progression-free survival prediction on a private dataset comprising 119 patients was described by Kickingereder et al. (2016). Positive findings with data-mining algorithms have also been reported when including Diffusion-MRI and relative cerebral blood volume data (Zacharaki et al., 2012) or Perfusion-MRI data (Jain et al., 2014) next to the MR-sequences used in the BraTS dataset. Deep learning based OS prediction has been successfully used on another, smaller (*n* = 93) private dataset (Nie et al., 2019). However, as the BraTS summary (Bakas et al., 2018b) indicates, deep learning techniques performed rather poorly on the open-access data. Quantitatively comparing deep learning to classical regression on radiomic features for OS on the BraTS data was also carried out by Suter et al. (2019). They concluded that radiomic feature are better suited, as features extracted from deep learning networks seemed to be unstable for this task.

Radiomic feature extraction describes the process of automatically computing a variety of quantitative image features. By quantifying lesions, radiomics can not only be used for prognosis, but can also help increase precision in diagnosis. For example, radiomics has been successfully used to distinguish between high- and low-grade glioma (Cho et al., 2018) on the BraTS dataset. An overview of radiomics and its applications is given by Rizzo et al. (2018). For brain tumor analysis in particular, a review of radiomics-based techniques for quantitative imaging is given by Zhou et al. (2018).

Radiomic features combined with a machine learning model is thus a natural choice for OS prediction. We initially evaluated different radiomics-based machine learning techniques for the BraTS challenge, too. However, when thoroughly validating the results, all considered approaches could not outperform a linear regressor based on the patients age only. We thus decided to submit an age-only linear regressor (Weninger et al., 2019), and won the third place in the BraTS challenge 2018.

In this paper, we analyze different radiomic-based approaches to survival prediction on the BraTS data. To be independent of segmentation inaccuracies, we only use the BraTS training data for all experiments. For this data, groundtruth segmentations are publicly available, approved by experts and reviewed by a single board-certified neuro-radiologist (Bakas et al., 2017c). The data can be subdivided by resection status into patients with reported GTR, subtotal resection (STR) and patients with unavailable resection status (NA). The official evaluation was carried out only on the GTR subset. First, we re-evaluate previously published radiomic signatures on the different resection status subsets. We show that these methods are predictive for OS on the STR subset. Second, different machine learning tools are evaluated on the radiomic feature set. Third, as the number of extracted radiomic features is very large and important features could remain undetected, two different feature reduction methods are assessed.

For the patients with GTR, neither previously published methods, nor different machine learning models, nor unsupervised feature reduction techniques could establish a robust signature for patient survival prediction. Finally, the importance of thoroughly assessing the robustness of radiomic markers is discussed, and ideas on how to improve survival prediction based on MRI images even after tumor resection are provided.

## 2. Materials

### 2.1. Dataset

In our evaluation, we discard the BraTS test- and validation datasets, as no groundtruth segmentations and no OS information are available, and use only the training dataset. All subjects of the BraTS 2018 dataset are included in the BraTS 2019 dataset; thus, the analysis is focused on the larger BraTS 2019 dataset. The BraTS survival data training dataset consists of data from 211 brain tumor patients from different institutions. For each patient, the following data is available:

- 4 MRI acquisitions: T1, T1 post contrast agent (T1CE), T2 and T2-FLAIR. All are resampled to an isotropic resolution of 1 × 1 × 1mm^3^, co-registered and skull stripped.
- Segmentation map: Edema (ED), enhancing tumor (ET), and non-enhancing / necrotic tumor core (NEC).
- The age of the patient.
- Resection status.

The resection status is either reported as GTR, subtotal resection (STR), or unknown (NA). For a few subjects (*n* = 21), the resection status was given as STR in the BraTS 2018 dataset, but omitted for the 2019 dataset. These statuses were re-entered into the dataset. Next, two patients were reported as still alive. Their overall survival in the database was set to the maximum survival time in the dataset, 1,767 days.

### 2.2. Cohort Study

Most data are provided either by the Center for Biomedical Image Computing and Analytics from University of Pennsylvania (CBICA, *n* = 128) or by The Cancer Imaging Archive (TCIA, *n* = 76) (Bakas et al., 2017a,b). A small amount of the data (*n* = 7) originates from other sources. All subjects have a pathologically confirmed diagnosis of primary *de novo* glioblastoma (Bakas et al., 2018b). Nevertheless, as population or differences in treatment could influence clinical outcome, an overview over differences and similarities of the different provenances is given.

**Figure 1:**
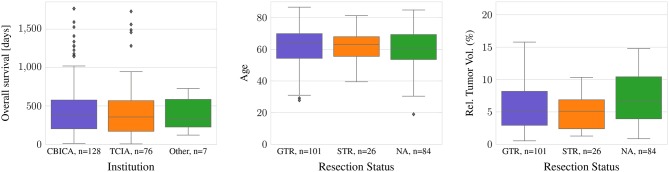
Difference in age and tumor-to-brain volume by resection status, and survival by provenance.

For all TCIA subjects, the resection status is unknown. In contrast, 94 of the 101 subjects with GTR as well as all subjects with STR originate from one institution, CBICA. In the dataset, there are no statistically significant differences between age or survival for the different data provenances or the different types of resection status (ANOVA: *p* >0.05). However, the relative brain tumor volume, as determined as tumor volume divided by brain volume, is significantly smaller in the TCIA data than in the CBICA data (*p* <0.0001). Between the resection status STR and GTR, in contrast, there is no significant relative brain tumor volume difference (Figure 1).

## 3. Methods

**Figure 2:**
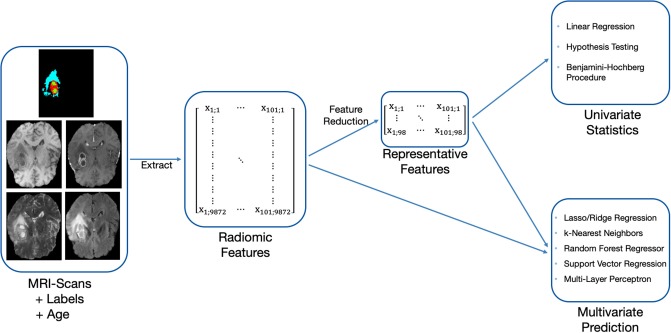
Methodology used to evaluate the predictiveness of radiomic features for OS with the available acquisitions and labels.

Our OS prediction pipeline can be divided into the following substeps: (1) Image preprocessing, (2) extraction of radiomic features, (3) unsupervised feature reduction, and (4) statistical inference and out-of-sample prediction. These major substeps of the pipeline are visualized in Figure 2. For the BraTS challenge, only out-of-sample prediction is necessary. In order to determine whether radiologic features are appropriate for the given problem, we supplement out-of-sample prediction with classical hypothesis testing.

### 3.1. Image Preprocessing

The data was acquired with various MRI scanners and different clinical protocols. In consequence, absolute image intensities, and, subsequently, radiomic features, can be strongly influenced. This was counteracted with a bias-filed correction and subsequent normalization of the images. First, the ANTs N3 (Tustison et al., 2010) bias-field correction was applied to all images, removing local differences in image intensities. Second, in order to harmonize the MRI acquisitions from different institutions, all images were normalized with z-score normalization to zero mean and unit variance.

Histogram equalization was considered as alternative normalization technique, but discarded as it did not improve the results. This could be due to the properties of tumor tissue in MRI images: Parts of the brain tumor are often the brightest or darkest area in the acquisitions, while occupying only a small proportion of the brain. The contrast-enhancing part is especially bright in T1CE acquisitions while covering just a small single-digit percentage of the brain volume. Histogram equalization or other nonlinear brightness adaptation techniques will thus shrink the contrast for these outlier points, actually leading to less contrast in the examined regions. For a comparison of the results using histogram equalization, all evaluations relying not only on tumor shape and/or age were repeated with histogram equalization instead of z-score normalization. The results can be found in the Supplementary Materials.

### 3.2. Feature Extraction

Using the package *PyRadiomics* (van Griethuysen et al., 2017), shape features were extracted from the provided segmentation masks, and image intensity and texture features were extracted from the four different image modalities for each segmentation mask. Image intensity and texture features were calculated for the original image and on wavelet decomposed images. In total, the following features were extracted:

**Shape features** comprise volume, surface area, sphericity, maximum diameter, elongation, axis lengths and flatness. These were extracted for the different tumor classes, resulting in 42 features.
**Gray-level features** include gray-level co-occurrence (glcm), gray-level run length (glrlm), gray-level dependence matrix (gldm), gray-level size zone, and neighboring gray tone difference features. As these were extracted for the original and wavelet transformed images and four image modalities, this resulted in 7,884 features.
**Image intensity statistics** consists of features such as minimum, maximum, mean, median, percentiles, standard deviation, skewness, kurtosis, and uniformity. In combination with different modalities and filters, 1,944 features resulted from this category.

Combined with the age, a total of 9871 features were obtained. In contrast, the total number of observations was 211—the number of variables *p* is much bigger than the number of samples *n*. Such a setting is actually common for pattern-learning methods in neuroscience (Bzdok, 2017), and is referred as *wide data*, in contrast to *long data* where the number of samples is bigger than the number of variables. Using such wide data directly for inference often leads to non-robust results and to overfitting on the training set. Consequently, before inference the number of features needs to be reduced as much as possible while maintaining the characteristics of the data.

### 3.3. Preselection of Features

Radiomic features are typically redundant (Rizzo et al., 2018), i.e., they are multicollinear. Different techniques exist to reduce the number of features and thus the multicollinearity. For the present problem, a subset of features should be kept after feature reduction. In contrast to synthetic features obtained by a PCA, a feature selection method offers more interpretable results. Further, in order to use the complete BraTS training dataset, the method should be unsupervised. With an unsupervised method, the complete BraTS OS training data (*n* = 211) can be used for feature selection, as features of preoperative images should be independent of resection status. In contrast, for this study, a supervised method could only be done on the specific resection status subset (GTR: *n* = 101). As splitting into train- and test set would further be necessary, an even smaller number of examples could be employed for feature selection.

**Figure 3:**
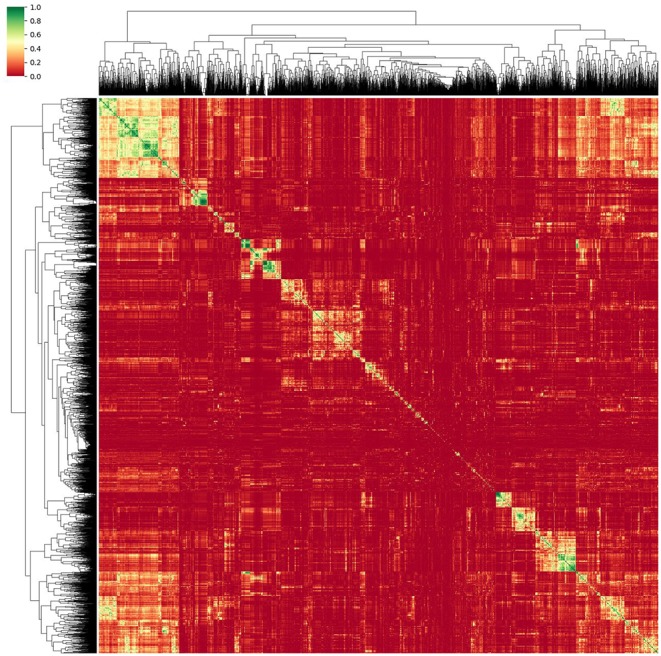
Reordered correlation matrix and obtained dendrogram of the radiomic features obtained from the normalized MRI images. Green designates strongly correlated features while red indicates an *r*^2^ close to zero.

Thus, a method relying on correlation matrix clustering and Variance-Inflation-Feature (VIF) iterative reduction (James et al., 2014) was chosen as the most appropriate. As a first step to reduce multicollinearity, single redundant features were discarded. For this purpose, each feature was linearly regressed against every other single feature, thus obtaining the coefficient of determination *r*^2^ and creating an *r*^2^ correlation matrix. This matrix was then reordered using a hierarchical clustering algorithm. For this, we relied on the Voor Hees Algorithm (Voorhees, 1986) implemented in SciPy (Jones et al., 2001) for linkage, and Euclidian distances between rows or columns of the correlation matrix. A visual impression of the obtained clustered correlation matrix is given in Figure 3.

As proposed by Gillies et al. (2016), representative features can be chosen from each cluster to reduce redundant elements. For this, areas of high correlation (*R*^2^ > 0.95) were reduced to the element with the highest inter-patient variability. Using this method, only features having a pairwise collinear correlation can be identified and omitted. Multicollinearity, i.e., highly related associations between more than two features, is not taken into account.

Multicollinear features were excluded in a second step. Those features can be identified by checking the VIF. Iteratively, by removing the feature with the highest VIF, the multicollinearity can be reduced until a predefined threshold is obtained. A maximum VIF of 10 is chosen, as thresholds of either 5 or 10 are recommended for this method (James et al., 2014). The number of features retained with a threshold of 10 should not pose problems to the machine learning models, so we did not consider lower thresholds.

Next to the VIF-based feature preselection method, we evaluated a principal component analysis (PCA) based feature reduction pipeline. One PCA feature reduction was carried out independently for the shape features, gray-level features and image intensity features of the original image. A fourth PCA was performed on all features of wavelet decomposed images. For each analysis, the minimum number of principal components explaining 95% of variance in the data were kept. The obtained features are finally concatenated, and the predictiveness for survival prediction can be evaluated via machine learning models.

### 3.4. Statistical Hypothesis Testing on Single Features

Null hypothesis testing with false discovery rate correction on the original dataset is not beneficial, as there are too many correlated features. The subset selected by the VIF feature selection (section 3.3), however, is much smaller and hypothesis tests can now reveal if single features are actually predictive for OS. As multiple radiomic features remained, a false discovery rate correction still needs to be used. We relied on the Benjamini–Hochberg procedure (Benjamini and Hochberg, 1995), that controls the false discovery rate at a specific level α = 0.05.

### 3.5. Multivariate Prediction

The statistical hypothesis testing can only reveal if single features are significantly predictive for OS. Nonlinear relationships of single predictors to the target variable as well as feature interactions cannot be detected. Different machine learning models that are able to surpass this limitation are available, ranging in complexity from basic linear regressors to complex neural networks.

We evaluated different machine learning models: linear, lasso and ridge regressors, k-nearest neighbors (kNN), random forests regressors (RFR), support vector regressors (SVR), and support vector classifiers (SVC). Furthermore, the Boruta (Kursa and Rudnicki, 2010) feature selection algorithm in combination with one random forest classifier (RFC) as estimator and one for the final prediction was evaluated. The regression models were directly fitted to the survival days, while the classifier can only predict the classes. As classes, the three classes as proposed by the BraTS challenge (*long-survivors* (OS >15 months), *short-survivors* (OS <10 months), and *mid-survivors* (10 months <OS <15 months) were used.

Different radiomic features are represented by absolute values at very different scales. Furthermore, outliers of single features may strongly influence the results. Consequently, the radiomic features were first normalized: The feature median is subtracted, and the features were scaled by the interquartile range, i.e., the range between the 25th quantile and the 75th quantile.

The different machine learning models were first employed on the complete feature set for the different resection status. The same methods were then also tested on the VIF-based feature subset as well as on the PCA reduced feature set, in order to evaluate whether these models could improve robustness on GTR patients.

All machine learning models were implemented with scikit-learn v0.21.2 (Pedregosa et al., 2011) or scikit-learn-contrib using default settings. Next to the methodology presented in this paper, we further evaluated the linear regressor on the age only as submitted during the BraTS challenge 2018, as well as a linear regression on age and the features remaining significant after Benjamini–Hochberg correction.

### 3.6. Evaluation of Previously Published Methods

Previously reported relationships between radiomic signatures and survival time were evaluated on the BraTS dataset. Gutman et al. (2013) reported that the length of the lesion's major axis and the proportion of contrast-enhanced tumor were negatively correlated with survival on the TCGA glioblastoma dataset. It should be noted that this dataset is included in the BraTS dataset with the resection status NA. It has also been shown that volumetric features of enhancing tumor, non-enhancing tumor core and necrosis, and edema normalized to brain volume are associated with shorter survival time on different independent datasets (Zhang et al., 2014; Macyszyn et al., 2015).

Kickingereder et al. (2016) proposed a supervised principal component analysis of radiomic features for glioblastoma patients. In this study, a set of MRI acquisitions also comprising diffusion and susceptibility-weighted MR imaging was used. Thus, compared to our analysis, the study relied on a different set of radiomic features. Nevertheless, their statistical analysis pipeline with z-score feature normalization and supervised principal component analysis is directly applicable to the features described in section 3.2.

## 4. Results

First, the predictiveness of state-of-the art methods and machine learning model using radiomic features is evaluated for the different types of resection status in sections 4.1 and 4.2.

**Figure 4:**
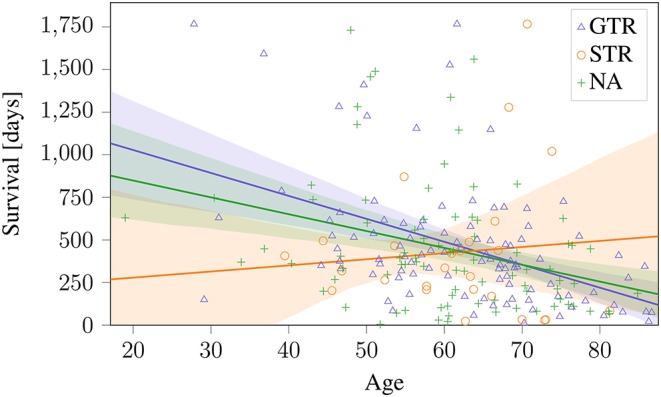
Age-only regression for the different types of resection status on BraTS 2019 training dataset. The obtained regression line is plotted together with the 95% confidence interval. This model is used as baseline against radiomics based approaches.

These sections show that a predictive radiomic signature can be extracted for STR patients. For these patients, radiomics based approaches to survival prediction outperform the age-only approach that can be seen in Figure 4. However, on the patients that underwent total resection of the tumor, the findings are different: The established radiomic features as well as all considered machine learning models fail to improve the survival prediction. Regression on age-only, however, is significantly correlated with shorter survival for GTR patients (Figure 4).

Then, as the results of different models show a high variability, it is assessed in section 4.3 whether models based on a selected subset of features can lead to more robust results for GTR patients.

### 4.1. Repeatability of Previous Methods on Dataset

As a first step, previously reported relationships between radiomic signatures and survival time (section 3.6) were evaluated on the BraTS dataset. For evaluation, the dataset was first divided by the three different reported types of resection status. The published radiomic signatures were evaluated on each subset individually.

**Figure 5:**
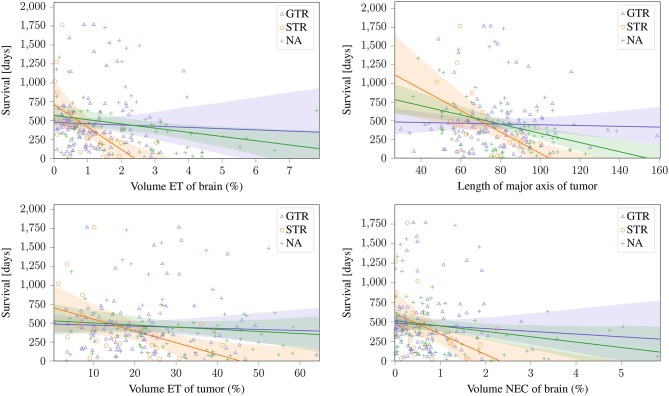
Linear relationship between previously reported significant features and the different resection status.

The two features proposed by Gutman et al. (2013) and the volumetric features of ET and NEC normalized to brain volume proposed by Zhang et al. (2014); Macyszyn et al. (2015) can be seen in Figure 5. These findings can be reproduced on the STR, and the same trends can also be seen on the NA subset (Pearson's r: *p* <0.05). Especially the ET volume and the lesions' major axis achieve a high significance (*p* ≈ 0.003) on the STR subset. Of the reported features, the only non-significant relationships are ED volume, that shows a negative, but non-significant (*p* >0.05) correlation on both subsets, and the ET tumor proportion, that shows a significant negative correlation on the STR subset, but only a non-significant negative correlation on the NA subset. However, on the subset with reported GTR, no correlation can be identified for any feature.

Next, the statistical analysis pipeline for radiomic features of glioblastoma patients proposed by Kickingereder et al. (2016) was applied to the different resection status subsets. In the original publication, MRI acquisitions that are not available in the BraTS data (e.g., diffusion MRI) and slightly different radiomic features were used. Nevertheless, the proposed z-score feature normalization and supervised principal component analysis is directly applicable to the present dataset, and can give a good baseline model. Using the proposed model parameters, the analysis was repeated on the radiomic features described in section 3.2 in a leave-one-out cross-validation. The results are compared to the age-only baseline approach for the different resections status in Table 1. It can be seen that the proposed supervised PCA approach achieves a higher accuracy and better mean square error than the age-only approach. In contrast, even as the age is included in the feature set, this approach fails on the GTR subset.

**Table 1:** Age-only linear regression compared to the supervised PCA (sPCA) model for the different types of resection status.

**Model**	**Accuracy**	**p (Binomial)**	**MSE**	**Median err**.	**SpearmanR**
**GTR**					
Age-only	0.48	0.00	109966	159	0.47
sPCA	0.27	0.20	148421	208	−0.41
**STR**					
Age-only	0.23	0.40	186772	194	−0.64
sPCA	0.46	0.21	148028	205	0.31
**NA**					
Age-only	0.37	0.49	136866	196	0.29
sPCA	0.26	0.20	159227	231	−0.03

The metrics are explained in section 4.2.

### 4.2. Multivariate Prediction

All methods were cross-validated in a leave-one-out setting, e.g., 100 samples were used to infer the 101th sample for the GTR dataset. From the 101 obtained results, the major test statistics as used in the BraTS challenge were computed: Accuracy (based on the three different time intervals described in section 1), mean squared error (MSE), median error, and Spearman rank correlation. For classifiers, all metrics are computed with respect to the class value (long-survivors: 824 days, mid-survivors: 379 days, short-survivors: 150 days). For the accuracy, we also assessed the statistical significance of the result with a binomial test and provide the p-value. All results can be seen in Table 2.

**Table 2:** Performance comparison of different machine learning models for the different types of resection status.

**Model**	**Accuracy**	**p (Binomial)**	**MSE**	**Median err**.	**SpearmanR**
**GTR**					
Regression	0.43	0.04	1778965427	394	0.11
Lasso	0.44	0.03	3070271	272	0.24
Ridge	0.42	0.07	1765220107	394	0.11
kNN	0.29	0.40	159451	248	−0.04
RFR	0.36	0.46	154447	190	0.14
SVR	0.27	0.20	140088	189	−0.77
SVC	0.41	0.11	175014	229	0.12
Boruta+RFC	0.45	0.02	133497	229	0.36
**STR**					
Regression	0.54	0.03	13748742	271	0.32
Lasso	0.46	0.20	415488	228	0.18
Ridge	0.54	0.03	13754344	272	0.32
kNN	0.50	0.09	173798	211	0.21
RFR	0.58	0.01	144744	125	0.43
SVR	0.23	0.40	175720	157	−0.65
SVC	0.31	0.99	221768	229	−0.43
Boruta+RFC	0.50	0.09	92744	229	0.45
**NA**					
Regression	0.32	0.91	1434873	412	−0.02
Lasso	0.39	0.25	533963	282	0.11
Ridge	0.32	0.91	1435941	412	−0.02
kNN	0.25	0.13	166072	254	−0.18
RFR	0.33	1.00	146459	247	0.20
SVR	0.25	0.13	155827	225	−0.78
SVC	0.40	0.16	169944	229	0.00
Boruta+RF	0.30	0.56	162734	229	−0.13

*All features as described in section 3.5 including the age were used as input. For the age-only approach as comparison, see Table 1*.

On the GTR subset, no model achieved better results than the age-only baseline. However, on the STR subset, most models were more predictive of survival than the age-only approach.

### 4.3. Feature Reduction Approaches

Several publications have shown the predictiveness of radiomics for survival prediction on different datasets (see section 1). On top, the re-implemented methods could reveal predictiveness of radiomic features for survival on patients with subtotal resection. However, these methods, as well as different machine learning models presented in this paper and as well as the majority of radiomic approaches submitted in the BraTS challenge 2018 failed on patients that underwent GTR. Thus, in this subsection, we focus on the GTR patients.

Even as all presented models performed worse than the age-only regressor, it can also be observed that the results of different machine models achieve strongly varying results. This could be due to the high number of radiomic features. Thus, it is evaluated whether the two proposed feature reduction techniques can produce more robust outcomes on the GTR dataset.

The presented unsupervised feature subset selection has two subsequent steps: First, the correlation matrix clustering, which suppresses pairwise correlated features, reduced the number of radiomic features from 9,870 to 5,338. Then, the VIF-based feature reduction, that checks also for multicollinearity, further reduced the number of features to 94. Combined with the age, 95 features were obtained, which is slightly less than the number of examples.

On this reduced feature set, hypothesis testing is feasible. Without any correction for false positives, 5 of the 95 features would have been considered significant (*p* <0.05). However, after Benjamini–Hochberg correction, only the age of the patient (*p* = 5.8 × 10^−5^) and one radiomic feature, the Wavelet LHH ImageIntensity Kurtosis on the necrotic part in the T2 acquisition remained significant. All statistically significant features can be seen in Table 3.

**Table 3:** Correlation analysis of VIF—selected features with OS for GTR patients.

**Feature**	**Correlation with OS**	***p*-value**
Age	−0.46	0.000001
Wavelet LHH ImageIntensity Kurtosis T2 NEC	0.39	0.00002
Wavelet LLL ngtdm Complexity T2 ET	0.22	0.03
Wavelet LHL ImageIntensity Kurtosis T1CE nec	0.22	0.03
Wavelet LLL ImageIntensity Minimum T1 ET	−0.20	0.05

Next, the unsupervised feature selection method based on PCA is considered. After applying PCA as explained in section 3.3, 15 principal components representing tumor shape were kept, 38 for the image intensity statistics features, 55 for the gray level features, and 98 for the wavelet features. The extracted features were concatenated together with the age in order to be used for multivariate prediction.

These features, as well as the features selected by the VIF-analysis, were separately employed for survival prediction (see Table 4). Consistent to 4.2, all features were normalized with a robust scaler, subtracting the median and scaling by the by the interquartile range, and the same machine learning models were utilized.

**Table 4:** Performance comparison of different feature selection methods and machine learning models for GTR patients.

**Model**	**Accuracy**	**p (Binomial)**	**MSE**	**Median err**.	**SpearmanR**
**VIF-BASED FEATURE SUBSET**					
Regression	0.47	0.01	28154236838	1,112	0.18
Regr. BH	0.46	0.07	109618	148	0.46
Lasso	0.36	0.60	2293591760	557	0.05
Ridge	0.33	1.0	16655918658	672	−0.02
kNN	0.30	0.53	159553	223	−0.08
RFR	0.35	0.75	149299	207	0.15
SVR	0.27	0.20	140181	189	−0.77
SVC	0.40	0.17	194331	445	0.06
**FEATURES EXTRACTED by PCA**					
Regression	0.39	0.17	672193	478	0.03
Lasso	0.36	0.59	688037	559	0.04
Ridge	0.38	0.25	558488	457	0.02
kNN	0.34	0.92	149014	194	0.07
RFR	0.34	0.92	163826	218	0.02
SVR	0.27	0.20	140298	189	−0.79
SVC	0.40	0.17	198655	445	0.05

*Regr. BH, Regression on all features that were significant after Benjamini–Hochberg multiple test correction*.

## 5. Discussion

Previous findings, especially those using volumetric features, could be reproduced for patients with subtotal resection. Furthermore, different considered machine learning models also showed predictiveness of survival. Thus, even as the sample size was limited, and different machine learning models show varying results, radiomic features seem to be correlated to patient survival for patients with subtotal resection.

However, when applying these methods to patients that underwent GTR, no significant relationship between radiomic features and overall survival could be identified. In effect, for this subgroup, the considered previously published and newly developed radiomic models could not identify any connection between image based features and survival that went beyond the predictiveness of patient age. In previously published findings, the resection status is often not known or not clearly stated (Gutman et al., 2013; Macyszyn et al., 2015; Kickingereder et al., 2016; Lao et al., 2017; Li et al., 2017), or radiomic features are not assessed dependent on resection status (Zhang et al., 2014; Nie et al., 2019). Patient age, a clinical marker that is not strongly predictive of survival for patient without total tumor resection (cf. Figure 4) seems to be the strongest predictor of patient survival after GTR. One single feature, the Wavelet LHH ImageIntensity Kurtosis T2 NEC, was statistically significant after Benjamini–Hochberg correction. However, after leveraging this finding in a predictive regression model, no clear benefit could be observed. Why radiomic features were not predictive on GTR patients remains unclear. It can only be hypothesized that survival for STR patients depends on the malignancy of the primary subtotally resected tumor, while survival for GTR patients relates to possible metastases that are not directly dependent on image features of the original tumor.

It can nevertheless be concluded that OS of brain tumor patients given radiomic images is strongly dependent not only on the preoperative images themselves. Given a high number of features and strong influences that cannot be assessed with preoperative MRI images, survival prediction is an ill-posed problem on a limited dataset. Researchers need to pay attention to the problems that arise when using radiomics or other big data methods on wide data, i.e., datasets with much more features than observations. Specifically, challenge participants and other researchers in clinical data analysis need to be fully aware of overfitting pitfalls, not only on the training set, but even on the validation dataset.

In radiomics, a very high number of features are extracted. In our case, a total of 9,871 features were initially considered. Combined with a limited dataset, as is often the case for medical applications, problems arise due to the curse of dimensionality. One problem encountered is the robustness of significance: The features that are significant on the whole dataset are not necessarily significant on the training subset, and vice versa, features identified as significant on a small dataset do not need to be significant on larger datasets. Although it is impossible to test all possible combinations of different radiomic features and machine learning models, we think that our evaluation shows the limitations of radiomic analysis on glioblastoma patients with GTR. To be as robust as possible against such subset biases, we used extensive cross-validation and determined an orthogonal subset of features.

Next to the difficulties encountered when applying radiomics to patients that underwent GTR, we believe one main limitation in the BraTS challenge 2018 was the small training dataset in combination with overfitted approaches. In the BraTS setting, a predefined validation set was released by the organizers, and could be used by all contributors to evaluate their algorithms during development. Thus, if contributors test different algorithms or hyperparameter settings on this left-out validation set, it has to be taken into account that one may accidentally overfit on this validation set. Such “result-peeking” invalidates accuracy scores on this left-out dataset, i.e. the developed approaches may generalize poorly to other samples. In fact, it seemed that this actually happened during the BraTS challenge. As can be seen on the official BraTS challenge online leaderboard (Bakas et al., 2018a), a total of nine different teams obtained accuracy scores at least as good as ours on the validation dataset. However, on the test set, our naïve algorithm scored the 3rd place out of 26 participants. On this dataset, segmentation and OS results could not be evaluated by participants, making this part of the data impossible to overfit. In contrast to private datasets, algorithm developers cannot—be it deliberately or accidentally—invalidate the obtained results by result-peeking in such a setting.

Thus, challenges such as the BraTS challenge are important for unbiased algorithm comparison and to assess whether findings from research are robust and can be applied to translational medicine. Here, it was assessed whether findings in radiomics of glioblastoma patients can be transferred to patients that underwent GTR. In this case, classical radiomic features seem not to be suited for robust results in survival prediction. In contrast, positive findings, with previously reported approaches as well as with different machine learning techniques can be reported for patients with subtotal resection.

Nevertheless, the approaches presented in this paper are not exhaustive. We do not want to present the new “best” survival prediction algorithm. Default parameter settings were utilized for all machine learning techniques, as exhaustive hyperparameter tuning—as employed by most winning approaches in machine learning challenges—on a small dataset would invalidate the results. The approaches presented in this manuscript, especially those relying on orthogonal feature subset selection, were utilized to analyze the robustness of radiomic features. They may not be the “best” algorithms for survival prediction. Thus, C-index, hazard ratio, or KM analysis were not regarded, as the focus of this analysis lies on robustness of radiomic features, and not on a single survival prediction algorithm.

## 6. Conclusion

The BraTS survival prediction challenge focuses on glioblastoma patients that underwent GTR. This paper shows that adding information from radiomic features to the age of the patient does not necessarily improve accuracy for this task. To show this, we evaluated different published techniques as well as a sophisticated radiomic feature extraction combined with modern machine learning techniques. However, no helpful information could be extracted, and our baseline—a linear regression on the age of the patient—could not be consistently outperformed on this limited dataset. In contrast, on patients with a different resection status—either where the resection status was not available or the tumor was subtotally resected—previously published findings could be reproduced, and different machine learning techniques could extract information predictive for overall survival.

In order to move from fundamental research to translational medicine, future research in brain tumor radiomics should focus on finding novel radiomic features that are applicable if the patient undergoes surgery. A possible set of features that was not assessed in this study are location based features. Location based features are not as established as shape or texture features in radiomics. However, they could be more promising for survival prediction even for patients that underwent GTR, as the position of the tumor in the brain could influence prognosis.

## Data Availability Statement

Publicly available datasets were analyzed in this study. This data can be found here: https://www.med.upenn.edu/sbia/brats2018/registration.html.

## Ethics Statement

This work relies only on the BraTS dataset. For the use of this dataset, no ethics statement is necessary.

## Author Contributions

LW performed algorithm development and implementation and wrote the manuscript. CH assisted in algorithm conception and interpretation of the results. DM supervised the work and critically revised the manuscript.

### Conflict of Interest

The authors declare that the research was conducted in the absence of any commercial or financial relationships that could be construed as a potential conflict of interest.
