# Supplementary material for: Robustness of Radiomics for Survival Prediction of Brain Tumor Patients Depending on Resection Status
Source: Front Comput Neurosci. 2019 Nov 8;13:73. doi: 10.3389/fncom.2019.00073 (PMC6857096; doi:10.3389/fncom.2019.00073)
Supplement: Supplementary file 1 [file Data_Sheet_1.pdf]

Supplementary Material

RESULTS USING HISTOGRAM EQUALIZATION

In the manuscript, the MRI images were normalized using z-score normalization. All experiments were repeated with histogram equalization instead of z-score normalization. The results of these experiments can be seen in the following tables. Experiments relying only on shape features and/or age were not repeated, as they are not affected by MRI image normalization.

Table S1. Age-only linear regression compared to the supervised PCA (sPCA) model for the different resection status. All results with histogram equalization instead of z-score normalization.

Model	Accuracy	p (Binomial)	MSE	Median Err.	SpearmanR
**GTR**					
Age-only	0.48	0.00	109966	159	0.47
sPCA	0.29	0.40	146696	159	-0.17
**STR**					
Age-only	0.23	0.40	186772	194	-0.64
sPCA	0.50	0.09	134484	157	0.58
**NA**					
Age-only	0.37	0.48	136866	196	0.29
sPCA	0.36	0.64	157699	222	0.08

Table S2. Performance comparison of different features and machine learning models for the different resection status. For the age-only approach as comparison, see Table S1. All results with histogram equalization instead of z-score normalization.

Model	Accuracy	p (Binomial)	MSE	Median Err.	SpearmanR
**GTR**					
Regression	0.42	0.07	2016088	283	0.07
Lasso	0.43	0.04	870320	293	0.08
Ridge	0.42	0.07	2015429	283	0.07
kNN	0.31	0.67	164095	226	0.07
RFR	0.39	0.25	144549	183	0.12
SVR	0.27	0.20	140532	191	-0.72
SVC	0.46	0.01	170227	229	0.23
Boruta+RFC	0.30	0.53	175650	445	0.03
**STR**					
Regression	0.46	0.21	695041	248	0.15
Lasso	0.38	0.54	296498	358	0.12
Ridge	0.46	0.21	695075	248	0.15
kNN	0.31	1.0	195174	261	-0.05
RFR	0.50	0.09	144336	151	0.36
SVR	0.23	0.40	175797	157	-0.68
SVC	0.46	0.21	151879	229	0.00
Boruta+RFC	0.58	0.01	60039	0	0.65
**NA**					
Regression	0.38	0.35	30491113282994	288	0.05
Lasso	0.37	0.49	600673	263	0.04
Ridge	0.36	0.64	30511661984726	308	0.06
kNN	0.31	0.73	164958	211	-0.10
RFR	0.30	0.56	139602	214	0.28
SVR	0.25	0.13	155547	225	-0.76
SVC	0.37	0.49	176600	229	-0.21
Boruta+RFC	0.26	0.20	157464	229	-0.16

Table S3. Correlation analysis of VIF-selected features with OS for GTR patients. All features with an uncorrected p-value <0.05 for Pearson correlation are given. After Benjamini-Hochberg multiple-test correction, only the age of the patient remains significant. All results with histogram equalization instead of z-score normalization.

Feature	Correlation with OS	p-Value
Age	-0.46	0.000 001
Original ImageIntensity Kurtosis T1CE ET	0.26	0.008
Wavelet LLL glrlm LongRunHighGrayLevelEmphasis Flair EDE	0.21	0.03

After applying PCA as explained in Section 3.3 to the histogram-normalized images, 15 principal components are kept to represent tumor shape, 39 for the image intensity statistics features, 18 for the gray level features, and 118 for the wavelet features.

Table S4. Performance comparison of different feature selection methods and machine learning models for GTR patients. All results with histogram equalization instead of z-score normalization.

Model	Accuracy	p (Binomial)	MSE	Median Err.	SpearmanR
**VIF-based feature subset**					
Regression	0.40	0.17	428482	287	0.14
Lasso	0.44	0.03	400681	274	0.16
Ridge	0.43	0.04	413358	277	0.15
kNN	0.37	0.46	160471	191	0.07
RFR	0.44	0.03	150711	204	0.18
SVR	0.27	0.20	139819	190	-0.73
SVC	0.45	0.02	161347	229	0.20
**Features extracted by PCA**					
Regression	0.42	0.07	417723	374	0.18
Lasso	0.37	0.17	420171	380	0.15
Ridge	0.41	0.11	359303	346	0.18
kNN	0.33	1.0	156324	188	0.05
RFR	0.43	0.04	141546	167	0.01
SVR	0.27	0.20	140285	189	-0.75
SVC	0.43	0.04	186603	445	0.13
